# Exosome-Based Therapeutics in Dermatology and Beyond: A Narrative Review

**DOI:** 10.3390/biomedicines14020338

**Published:** 2026-02-01

**Authors:** Grant M. Pham

**Affiliations:** Dermatology, Arlington Regenerative Medicine Institute, Arlington, TX 76013, USA; grantmpham@gmail.com

**Keywords:** exosomes, extracellular vesicles, dermatology, aesthetics, wound healing, alopecia, photoaging, scars, osteoarthritis

## Abstract

Exosomes are small extracellular vesicles that package DNA fragments, several classes of RNA, lipids, and proteins, and are now regarded as active messengers between cells rather than as cellular debris. This narrative review synthesizes dermatologic and related regenerative applications reported between 2020 and 2025, drawing on PubMed and Scopus searches. In skin, exosomes regulate inflammation, angiogenesis, matrix remodeling, pigmentation, and hair cycling. Preclinical models show faster wound closure, improved scar architecture, attenuation of photoaging changes, and stimulation of hair growth, with additional signals in inflammatory dermatoses and fungal skin disease. Early human studies in wound care, rejuvenation, scars, and alopecia suggest acceptable safety and a recurring pattern of benefit when exosomes are used as adjuncts to microneedling, lasers, or standard dressings, although products, dosing, and outcome measures remain heterogeneous. Beyond dermatology, early work in osteoarticular and soft tissue repair points toward meaningful regenerative potential, but clinical programs are still at an early stage. In practice, exosomes are being positioned as acellular alternatives or add-ons to platelet-rich plasma, bone marrow aspirate concentrate, and conventional topicals and as emerging carriers for small molecules and biologics. Key limitations include low yields, product and cargo heterogeneity, lack of agreed quality and potency metrics, and uncertain regulatory status. Whether exosomes remain boutique adjuncts or become part of standard dermatologic and musculoskeletal practice will depend on what happens next: consistent manufacturing, agreed-upon characterization panels, meaningful potency assays, robust pharmacokinetic and biodistribution data, and comparative trials that track outcomes and safety over years rather than weeks.

## 1. Introduction

Extracellular vesicles encompass exosomes, which are the smaller endosome-derived particles, and larger microvesicles that bud from the plasma membrane [1]. Once dismissed as acellular waste, they are now understood as messengers that carry nucleic acids, proteins, and lipids capable of reshaping the behavior of recipient cells. Cutaneous tissues rely on vesicles from keratinocytes, melanocytes, fibroblasts, endothelial cells, and immune cells. These in turn organize inflammatory responses, rebuild the barrier, remodel the matrix, and regulate pigment and follicular cycling. In osteoarticular and peri-tendinous tissues, mesenchymal and stromal cell vesicles deliver similar paracrine cues to chondrocytes, synoviocytes, and tenocytes, helping to maintain joint integrity and guide repair after damage [2]. These insights have prompted growing interest in therapeutic use of exosomes as cell-free biologics that can harness paracrine signaling without the logistical and safety challenges of live cell therapy. In dermatology, most attention has focused on wounds, scars, photoaging, alopecia, inflammatory dermatoses, and more recently on skin infections such as fungal disease. Parallel but earlier work is emerging in osteoarthritis and tendon or enthesis repair as clinicians search for minimally invasive options for an aging population. This review concentrates on exosomes as practical tools rather than only as biomarkers, with emphasis on mechanisms of action, manufacturing and formulation, clinical use around procedures such as microneedling and lasers, safety and regulatory issues, and the major evidence gaps that must be addressed before exosome-based products can be integrated confidently into routine cutaneous and musculoskeletal care (Table 1).

## 2. Materials and Methods

This narrative review draws on studies published between 2020 to 2025 that are indexed in PubMed and Scopus, with additional trial records identified on ClinicalTrials.gov. Both preclinical and in vitro animal experiments and human studies were included. Human data ranged from case reports/series to prospective cohorts to randomized controlled trials. Greater weight was given to level I and II evidence when it was available. Studies were eligible if they used therapeutic extracellular vesicles or exosomes in dermatology. These included cosmetic/aesthetic practice, wound care, hair and scalp disorders, inflammatory dermatoses, or osteoarticular disease. Also, they were included if they reported at least one biological or clinical outcome. Commentary pieces and reports that did not clearly describe an intervention and outcome were excluded. Searches were run in each database using combinations of controlled vocabulary and free text. The core exosome terms were (exosome* OR “extracellular vesicle*” OR “small extracellular vesicle*” OR “EVs” OR “sEVs”). These were combined with skin-focused terms: (skin OR cutaneous OR dermatology OR dermatologic OR cosmetic OR aesthetic OR “wound healing” OR wound* OR ulcer* OR scar* OR keloid* OR “photoaging” OR “skin rejuvenation” OR alopecia OR “hair loss” OR psoriasis OR “atopic dermatitis” OR eczema OR acne) and with musculoskeletal terms: (osteoarthritis OR cartilage OR chondrocyte* OR joint* OR synovitis OR tendon* OR enthesis OR “tendinopathy”). An example of a full string included (exosome* OR “extracellular vesicle*”) AND (skin OR cutaneous OR dermatology OR cosmetic OR aesthetic); (exosome* OR “extracellular vesicle*”) AND (wound* OR ulcer* OR scar* OR “photoaging” OR alopecia OR “hair loss”); and (exosome* OR “extracellular vesicle*”) AND (osteoarthritis OR cartilage OR tendon* OR enthesis). Titles and abstracts were screened, full texts were reviewed by the author, and the reference lists of key articles and recent guidelines such as MISEV2023 were scanned to identify additional relevant studies.

## 3. Biology and Mechanisms of Action

### 3.1. Biogenesis and Cargo

Extracellular vesicles include exosomes, usually about 30 to 200 nm in diameter, which form by inward budding of endosomal membranes. This outward budding produces microvesicles, typically 50 to 1000 nm across, that detach straight from the plasma membrane. Each one contains a slice of the parent cell’s molecular content: DNA fragments, several classes of RNA, proteins including enzymes and receptors, and bioactive lipids. This molecular cargo mirrors the functional state of the source cell and can reshape the behavior of recipient cells through paracrine signaling [1]. Contemporary consensus and reviews highlight ESCRT/tetraspanin- and lipid-mediated cargo sorting and emphasize that these payloads modulate inflammation, matrix remodeling, and regeneration across tissues [3].

### 3.2. Pathway Modulation (MoA)

Across cutaneous and musculoskeletal contexts, exosome cargo modulates several repair pathways. It can dampen inflammatory signaling, particularly through the nuclear factor kappa B (NF κB) and signal transducer and activator of transcription (STAT) pathways. These pathways, which sit near the center of many cytokine and stress responses, shift macrophages toward pro-resolving phenotypes and fine-tune T-cell activity. In remodeling, exosomes regulate TGF-β/SMAD signaling, influence myofibroblast transition, and rebalance the MMP/TIMP axis to improve collagen I/III organization [4]. They also promote angiogenesis and re-epithelialization via pro-vascular miRNAs and growth factor programs that enhance endothelial migration, tube formation, and keratinocyte–fibroblast proliferation [5]. In pigmentary and hair biology, exosomes can alter melanogenesis through MITF/tyrosinase pathways and stimulate anagen entry by reinforcing Wnt/β-catenin exchange between dermal papilla cells and follicular stem cells [5].

### 3.3. Pharmacokinetics and Biodistribution

Exosomes show route-dependent kinetics after local administration: topical or intradermal placement favors local tissue retention but must overcome the stratum corneum, for which strategies like microneedles and hydration/penetration enhancers improve delivery. Direct skin penetration of free sEVs is shallow without such aids [4,6,7]. In joints, intra-articular dosing yields synovial residence with therapeutic signals in osteoarthritis models, and residence/uptake can be augmented by viscoelastic carriers (for example, hyaluronic acid (HA) gels) or surface engineering (e.g., charge-reversal) to mitigate rapid clearance [8,9]. Across delivery routes, following exosomes in vivo is still not straightforward. Many commonly used fluorescent dyes can create their own particles, so the signal does not always reflect vesicles themselves. Recent method papers therefore argue for more careful labeling and for combining several imaging approaches when describing biodistribution and pharmacokinetics [10].

## 4. Manufacturing and Quality

### 4.1. Exosome Sources and Upstream Production Variables

Therapeutic exosomes are usually produced from human cells, most often mesenchymal stromal cells from bone marrow, adipose tissue, or umbilical cord. Dermal fibroblasts and keratinocytes are also used, and each cell type generates vesicles with a distinct cargo and pattern of activity that is relevant to skin repair and regeneration [11]. To make production more scalable and affordable, investigators are testing non-human sources. Vesicles from bovine colostrum have shown benefits in experimental models of atopic dermatitis, and plant-derived vesicles that behave like exosomes are being developed for topical and cosmetic use as simple biocompatible carriers [12]. Clinically relevant exosome output depends not only on the starting cell type but also on how those cells are grown and the way the bioreactor is configured, since these factors shape yield, shelf life, and potency.

### 4.2. Isolation and Characterization

Exosome preparations are most often separated by differential ultracentrifugation, tangential flow filtration, and size-exclusion chromatography [13]. Tangential flow filtration is gaining favor because it scales more easily and shortens processing time. It also tends to lower protein carryover compared with ultracentrifugation. For these reasons, it is now being built into good manufacturing practice (GMP)-style manufacturing workflows [13]. Size exclusion chromatography tends to give gentler, higher purity fractions, but both recovery and cargo composition depend strongly on the column and how the system is set up [14]. According to the most recent Minimal Information for Studies of Extracellular Vesicles consensus (MISEV2023), exosome characterization should not rely on a single assay. It should report particle size and counts, typically with nanoparticle tracking analysis, and document vesicle morphology. MISEV2023 also recommends a protein panel that includes tetraspanins such as CD9, CD63, and CD81 together with other vesicle-associated proteins like TSG101 and ALIX, plus negative markers to show that common contaminants have been removed [3]. In addition, functional potency assays that match the intended clinical indication, for example wound closure, angiogenesis, or immunomodulation bioassays, are recommended alongside analytic characterization.

### 4.3. Yield, Formulation, and Stability

Exosome manufacturing still suffers from low yields per culture, so many groups are turning to microcarrier-based and other bioreactor systems, three-dimensional culture, and related upstream changes to increase vesicle release [15,16]. Biostimulation is also being explored. Low level light or laser exposure can increase EV secretion through Wnt-related pathways in vitro and is discussed in contemporary wound repair reviews [17]. For delivery, hydrogels, creams, and buffered injectable formulations are common, and hydrogels in particular improve local retention and provide sustained release in skin and wound models [18,19]. Stability remains a concern, and cold chain requirements have prompted lyophilization strategies and storage best practice reviews, with encouraging reports of room temperature formulations for milk derived and plant derived vesicles [20,21]. When compared with platelet-rich plasma (PRP), platelet-rich fibrin (PRF), or living cells, EVs are generally seen as more compatible with storage and appear to have low, although not absent, intrinsic immunogenicity, which makes them attractive as drug carriers able to transport RNAs, proteins, and small molecules [22,23,24].

### 4.4. Standardization and Release Criteria

Exosome products remain highly heterogeneous because they are derived from different source cells and are manufactured under varied culture and separation conditions, and there is still no broadly agreed upon reference product or benchmark preparation. The most recent consensus guidance, MISEV2023, together with several regulatory analyses, recommends that developers define a clear set of critical quality attributes for exosome products. These attributes should cover identity and basic physicochemical characterization, including particle size and counts, morphology, and expression of canonical EV markers. They should also address purity and the removal of co isolated contaminants and include functional potency assays that match the intended clinical indication. In addition, stability data that reflect storage and transport conditions are needed [3,25]. Recent reviews from 2024 and 2025 repeat these themes and note that regulators currently regard exosome preparations as unapproved biologic drugs, which means they are expected to meet the same quality standards as other biologic products [25,26].

### 4.5. Compliance and Facilities

Compliance and facility standards remain a major concern. In the aesthetic space, exosome products are sometimes advertised with therapeutic claims even though they are prepared and dispensed outside of GMP environments, which raises the risk of contamination, misbranding, and poor traceability. The United States Food and Drug Administration (FDA) has repeatedly stated that no exosome products are currently approved and that any product promoted for treatment is regulated as a drug or biologic, requiring an IND or BLA and current GMP manufacturing; the agency has issued consumer safety notices and multiple warning letters to clinics and suppliers on this basis [25]. The FDA also makes clear that when a cosmetic product is marketed with claims to diagnose, treat, or prevent disease, it is considered a drug under United States law and must be labeled and regulated accordingly [25]. In Europe and the United Kingdom, extracellular vesicle therapeutics may fall within the advanced therapy medicinal product framework, which carries specific expectations for quality, pharmacovigilance, and traceability [27]. Across regions, and in line with MISEV2023, there is a strong push to protect patients and align clinical use with regulatory expectations by tightening manufacturing standards, improving labeling accuracy, and ensuring batch-level traceability.

## 5. Evidence in Dermatology

### 5.1. Wound Healing (Acute/Chronic)

Wound healing applications are supported mainly by preclinical data and a smaller but steadily growing number of human studies. In animal and in vitro models, extracellular vesicles from mesenchymal, adipose, bone marrow, and epidermal sources accelerate re-epithelialization, increase granulation tissue and angiogenesis, improve collagen organization, and reduce inflammatory mediators such as TNF alpha and interleukin 1 beta [28,29,30]. Recent systematic reviews and meta-analyses of stem-cell-derived exosomes largely confirm these findings and report benefits across the inflammatory, proliferative, and remodeling phases of repair. However, protocols, dosing strategies, and delivery systems remain highly variable [31,32].

Across wound types, diabetic foot ulcers have received the most attention. Meta analyses of preclinical work indicate that bone marrow stem cell vesicles and adipose stem cell vesicles enhance closure, neovascularization, and collagen deposition in diabetic and non-diabetic wounds [33]. Early human experience is emerging. A recent case series using topical adipose-derived stem cell exosomes for chronic lower extremity ulcers reported progressive area reduction and improved vascular parameters without serious adverse events, while another case series described faster healing after surgical and aesthetic procedures with topical mesenchymal cell exosomes in an office setting [34,35]. Larger controlled trials are now registered for diabetic foot ulcers and difficult wounds, but most have not yet reported outcomes.

Other biostimulatory modalities help frame expectations for exosome use. Evidence from randomized studies and meta-analyses indicates that low-level light-based therapies can help wounds close faster and lessen pain in conditions such as chronic ulcers, partial thickness burns, and pressure sores. However, investigators have used different wavelengths, doses, and devices, which makes the results somewhat heterogeneous [36,37]. As a growth-factor-rich treatment, PRP has been the subject of multiple new meta-analyses in diabetic foot and venous leg ulcers. Most report better healing rates and faster closure when PRP is added to usual care. Nonetheless, differences in PRP protocols and methodological quality introduce considerable heterogeneity [38,39]. Together, these data give credence that exosomes fit within a broader biostimulatory model of wound management, but confirm that rigorously controlled trials with standardized products/outcomes are still needed before widespread adoption.

### 5.2. Photoaging and Rejuvenation

In photoaging, exosome treatments are linked to dermal remodeling with increased collagen and elastin markers, which result in better matrix organization and reduced oxidative and inflammatory signaling in preclinical models [40]. Early clinical data largely come from split-face and pilot studies where exosomes are layered on top of device-based rejuvenation. In a twelve-week split-face trial by Park et al. (2023), microneedling plus adipose stem-cell-derived exosomes improved hydration, elasticity, and pigmentation more than microneedling alone, with benefits that became evident by six weeks [41]. A more recent investigator-blinded split-face study compared exosomes with PRP delivered with radiofrequency microneedling for facial photoaging. Both arms improved wrinkles, dyschromia, erythema, texture, and global appearance. The histology had increased collagen and glycosaminoglycans, but there was no clear superiority between the two biologic arms [42].

Fractional laser-based protocols show similar findings. A prospective study by Kwon et al. (2020) performed a fractional carbon dioxide laser followed by topical adipose stem cell exosomes and reported better texture and dyspigmentation scores along with shorter downtime on the exosome-treated side [43]. Subsequent pilot work and observational split-face series, grouped under the term laser-assisted exosome delivery, describe enhanced rejuvenation and faster recovery when exosomes are applied after fractional carbon dioxide or combined with microneedling in full-face protocols, although patient numbers are small and designs are uncontrolled [44]. A recent aesthetic review by Rodriguez et al. (2024) emphasized that products, doses, and outcome measures are heterogeneous [45]. Expectations should remain modest until larger randomized trials with longer follow up and standardized endpoints confirm these early findings.

### 5.3. Scars (Hypertrophic, Atrophic) and Procedural Recovery

Small human series (including plant-derived exosome products used alongside standard care) describe improved texture, pliability, and cosmetic outcomes. However, the series were uncontrolled [46]. In scarring, early clinical and preclinical work suggests that exosomes can improve scar quality and shorten recovery, especially when used as adjuncts to procedures. A double-blind randomized split-face trial found that topical adipose mesenchymal stem cell (MSC) exosomes applied after fractional CO_2_ resurfacing for atrophic acne scars produced greater improvements in validated scar scores and faster recovery than the control on the opposite side, with only mild local reactions reported [47]. Other small procedural series describe similar patterns of earlier reepithelialization and better texture when exosomes are layered onto fractional laser or microneedling protocols, but these studies are short, single center, and often lack a true comparator [48,49].

Experimental studies in hypertrophic and keloid scars report that selected EV preparations can modulate fibroblast activity, collagen organization, and inflammatory pathways, leading to anti-fibrotic effects in animal models [50]. At the same time, vesicles derived from keloid fibroblasts themselves appear pro-fibrotic, which underscores the need for careful source selection and product characterization before clinical use. Small human series, including those that use plant-derived exosome-like products alongside standard care, describe better texture, pliability, and cosmetic appearance, but these reports are uncontrolled and at risk of bias [46]. Overall, the signal for scar and procedural recovery is encouraging, yet robust randomized controlled trials with standardized scar scales and transparent reporting are still needed before exosomes can be recommended as routine scar therapy.

### 5.4. Alopecia and Hair Biology

In hair biology, exosomes appear to support communication between dermal papilla cells and follicular stem cells and can help push follicles toward the anagen growth phase. This effect is linked mainly to activation of Wnt and β-catenin signaling and to modulation of perifollicular inflammation that contributes to miniaturization [51,52,53]. Dermal papilla-derived vesicles carrying miRNAs such as miR-181a-5p or miR-218-5p, as well as exosomes from umbilical cord and other mesenchymal sources, increase hair follicle proliferation and prolong anagen in preclinical models through Wnt/β-catenin and PI3K/Akt pathways [54]. PRP-derived exosomes add another layer of support by enhancing follicle growth via the same Wnt/β-catenin axis, which helps connect exosome biology to more established regenerative tools. A recent systematic review by Gupta et al. (2023) synthesized these data and concluded that mesenchymal and dermal papilla exosomes are plausible cell-free candidates for alopecia treatment but emphasized that most evidence remains preclinical or early phase [55].

Early human experience is growing but still modest. A prospective study of patients with androgenetic alopecia who received intradermal injections of a standardized allogeneic exosome product reported decreased shedding and improved hair density and shaft diameter at 4 and 12 weeks, with only transient local reactions [56]. Furthermore, a systemic review by Al-Ameer et al. (2025) showed most exosome hair regrowth protocols used a short series of in-office treatments over 2 to 3 months or a single procedure with follow up of 6 to 12 months, but none defined a long-term maintenance schedule comparable to the daily, indefinite use expected with minoxidil or finasteride [57]. Case series using autologous and “humanized” exosomes for pattern hair loss describe visible thickening and increased density on trichoscopy, but protocols, dosing, and follow-up vary widely [53]. Procedural combinations are a recurring theme. Microneedling or microneedling radiofrequency followed by topical exosomes has shown gains in hair density and diameter in small prospective cohorts, along with high patient satisfaction and minimal downtime, yet these studies are single center and often lack robust control arms [58]. Recent scoping and comparative reviews suggest that exosome-based approaches may perform at least comparably to PRP and, in some analyses, better than topical minoxidil, but they also stress the heterogeneity of products and endpoints and call for properly powered randomized trials [57].

Case reports describe complete hair regrowth in alopecia areata after treatment with rose stem-cell-derived exosomes, with restoration of natural hair color and no major adverse events [59]. Several clinical trials are now registered to test plant or cosmetic exosome formulations in male pattern hair loss, but most are ongoing and have not yet reported outcomes. Overall, the hair data support a promising trend for clinical expansion. However, it reinforces the need for standardized products, dosing schedules, and endpoints before exosomes can be integrated into routine alopecia care.

### 5.5. Inflammatory Dermatoses

Inflammatory dermatoses show convergent EV biology centered on immune modulation and barrier repair. In atopic dermatitis (AD), keratinocyte-derived EVs from filaggrin-deficient skin promote type-2 skewing by activating CD1a-restricted T-cell responses and amplifying IL-4/IL-13 signaling [60]. In contrast, MSC-EVs reduce itch and Th2 signaling while restoring filaggrin/claudin expression and lipid-barrier enzymes [61]. Early translational work suggests that combining EVs with emollients or topical anti-inflammatories can further improve eczema severity [62].

In psoriasis, resident skin-cell and immune-cell EVs carry miRNAs and proteins that drive NF-κB/IL-23–Th17 pathways, thus fueling keratinocyte hyperproliferation [63]. Candidate therapeutic EVs counter this by rebalancing Th17/Treg, lowering TNF-α and IL-1β, and thereby normalizing keratinocyte differentiation markers [64]. There are several studies that link circulating EV cargos (e.g., PD-L1, specific miRNA panels) with disease activity, highlighting biomarker potential [65].

In acne, a condition that affects approximately 85% of the world’s population, microbiome-derived EVs especially from *Cutibacterium acnes* activate TLR2 on keratinocytes and immune cells, escalating NF-κB/NLRP3 signaling and IL-1β release [66,67]. This amplifies perifollicular inflammation, hyperkeratinization, and sebum dysregulation that drive comedogenesis as seen in Table 1. By contrast, candidate therapeutic EVs (e.g., MSC-derived or engineered EVs) have been shown to attenuate TLR2 pathways, lower pro-inflammatory cytokines, and normalize sebocyte lipogenesis in human skin models [68]. Early translational work is exploring topical gels and microneedling-assisted delivery with practical endpoints such as inflammatory lesion counts, GAGS, and sebumetry [69]. Overall, EVs look promising as biomarkers and adjunctive treatments, but standardized products, dosing, and trial endpoints are still needed before routine clinical use.

### 5.6. Skin Infections and Fungal Disease

Work on extracellular vesicles in infectious skin disease is still early, but it points to an important role for vesicles in host–pathogen interactions. Fungal pathogens, including *Candida* and *Malassezia* species, release vesicles that carry enzymes, lipids, nucleic acids, and allergens [70]. These vesicles can shape virulence, biofilm behavior, and host immunity and are now recognized as part of the fungal “tool kit” in infection [71]. In *Candida albicans*, biofilm-derived vesicles deliver virulence factors and toxins to host cells, influence drug responses, and contribute to invasive disease in experimental systems [72]. These observations are mainly mechanistic, but they suggest that targeting fungal vesicle pathways could complement standard antifungal therapy in the future.

Therapeutic concepts are only beginning to appear. Most work so far uses vesicles as novel delivery systems for antifungal drugs rather than as active biologics on their own [73]. A recent example is a cucumber-derived plant vesicle system loaded with terbinafine, designed as a transdermal formulation for superficial fungal infection; this platform improved skin penetration and local drug exposure in preclinical models and reduced fungal burden compared with free drugs [74]. This approach could lessen the need for invasive nail procedures for onychomycosis and may also decrease reliance on prolonged courses of systemic antifungal therapy, which carry a well recognized risk of liver toxicity [75]. Similar plant- or milk-derived vesicles are being explored as topical carriers with antioxidant and anti-inflammatory properties that might support barrier repair while delivering conventional antifungals [76,77]. At present there are no controlled human trials of exosome-based therapies for cutaneous fungal infections, and fungal vesicles themselves likely act as disease drivers rather than treatments. The near-term research need is therefore two-pronged: one is needed to better define how pathogen-derived vesicles contribute to skin infection and second to test host- or plant-derived vesicle platforms as safer vehicles for antifungal drugs in rigorously designed studies.

## 6. Beyond Dermatology: Osteoarticular Applications

### 6.1. Cartilage and Osteoarthritis

In knee osteoarthritis, a commonly used regenerative products used for healing and improvement is PRP. Meta analyses show small to moderate improvements in pain and function versus placebo and HA at up to twelve months, but results vary by preparation and dosing, and leukocyte-poor products often perform better [78]. Also in knee osteoarthritis, animal studies consistently show that intra-articular exosomes protect cartilage, calm synovial inflammation, and improve pain-related behavior and histology. Recent meta-analyses pooling preclinical models reached the same conclusion and supported a disease-modifying signal in knees [79].

Human evidence is still early and lacking. A triple-blind randomized trial by Bolandnazar et al. (2024) that injected placental MSC-derived extracellular vesicles into one knee and saline into the contralateral knee found the product was safe but not superior to the placebo on symptoms or MRI at six months [80]. Thus, to date there are no exosome treatment studies that outperform established options such as PRP or HA. Additional phase 1 and early phase 2 trials of MSC small EVs are registered, with results pending.

The clinical need is large and growing stronger than ever. Global Burden of Disease projections estimate roughly 642 million people will be living with knee osteoarthritis by 2050, driven by population aging and obesity [81].

### 6.2. Tendon and Enthesis

Preclinical work suggests that extracellular vesicles can aid tendon repair and enthesis healing. In Achilles models, mesenchymal stem cell vesicles improve histology, collagen organization, mechanical strength, and pain-related behavior. They also shift macrophages toward pro-resolving states. Evidence extends to tendon–bone interfaces [82]. Studies in rotator cuff and anterior cruciate ligament models show better fibrocartilage formation and stronger attachment after treatment with stem cell exosomes or platelet-derived exosome preparations, especially when combined with supportive biomaterials that improve local retention [83,84]. Recent systematic reviews reached similar conclusions and called for standardized products, dose finding, and rigorous reporting [85]. Human trials are still lacking, so translation will depend on early phase studies that confirm safety and define clinically meaningful endpoints for tendon healing [86].

### 6.3. Quality of Evidence

Taken together, the current dermatology literature is dominated by small, single-center, often uncontrolled studies with short follow up and heterogeneous products, dosing, and outcome measures. Many reports use proprietary preparations with incomplete characterization, and risk of bias is difficult to assess. These limitations make it hard to compare results across studies or to define best practice protocols, even where signals of benefit are encouraging.

## 7. Clinical Translation and Practice Considerations

In aesthetic clinics, exosomes are usually positioned as adjuncts to procedures rather than as standalone treatments. Good candidates are patients seeking recovery support after microneedling or lasers or those with photoaging or scars who already plan a device session. Counseling should include regulatory status, product provenance, and the limited human evidence base.

Protocol integration is evolving. Split-face studies report that topical adipose-derived exosomes after fractional CO_2_ improved scar and texture outcomes compared with control care and that pairing exosomes with microneedling can improve photoaging measures [41]. These data support peri-procedural timing that places exosomes immediately post-device to leverage microchannels for uptake. Dose metrics differ by product; most reports track particle counts or protein load, but no clinical standard exists. Comparative conversations should include PRP and HA, since both are established procedural adjuncts.

For ongoing practice, prioritize standardized outcomes. Use validated scales for scars and photoaging, and record adverse events and recovery time. Recent dermatology reviews summarize these approaches and highlight the need for controlled trials before routine adoption.

From a regulatory standpoint, most exosome preparations used in clinics are best regarded as investigational biologic products rather than licensed therapies. They have not undergone full agency review for safety, efficacy, and manufacturing quality, and many are produced in settings that may not meet all GMP requirements. In addition, practical issues such as scalability, batch to batch consistency, cost of manufacture, and patient-level affordability are still largely unresolved. Together these factors will influence whether exosome products remain niche interventions in select clinics or eventually become standardized treatments with broader access.

## 8. Future Directions and Research Priorities

Translation of exosome-based therapeutics will depend on resolving three interlocking domains: product quality, pharmacology, and clinical evidence. At the manufacturing level, alignment with MISEV-style principles and emerging regulatory guidance will require formal definition of critical quality attributes including but not limited to particle metrics, marker panels, purity thresholds, and indication-specific potency assays. Second is to establish basic pharmacokinetic and pharmacodynamic profiles. Rigorous tracking studies are needed to define absorption, tissue distribution, and persistence. Investigators should define how exosomes are handled by the body after topical, intradermal, and intra-articular delivery. Third, exosome products should be tested in well-powered registration-ready trials that use active comparators, have standardized outcomes, and are linked to long-term safety registries.

## 9. Conclusions

Exosomes modulate inflammatory, matrix remodeling, pigmentary, and follicular pathways. In cutaneous models, this activity leads to faster reepithelialization, more organized collagen deposition, and lower levels of inflammatory mediators. Similar patterns are beginning to appear in small early studies of photoaging, scarring, and androgenetic alopecia, although sample sizes are modest and protocols vary. In contrast, musculoskeletal repair remains largely preclinical, and the one triple-blind randomized trial in knee osteoarthritis has shown an acceptable safety profile but no advantage over saline. Taken together, these data support a coherent biological rationale, but the overall clinical evidence remains limited and incomplete.

Overall, exosome therapies are still in their early stage. Basic questions about product identity, potency, and dosing are only beginning to be answered. Most available studies are small or uncontrolled. Yet the same features that make exosomes biologically attractive in the lab could eventually make them useful in the clinic. As manufacturing becomes more consistent and comparative trials accumulate, it should be possible to define where exosomes genuinely add value alongside or beyond existing options such as PRP, HA, and energy-based procedures. Until then, they are best regarded as investigational tools with promise rather than established treatments.

## Figures and Tables

**Table 1:** Summary of exosome applications in cutaneous indications.

Indication	Main Targets and Mechanisms	Typical Exosome/EV Sources (Cell Origin)	Characterization Commonly Reported in Clinical or Near-Clinical Studies	Typical Treatment Schedule Reported in Clinical Studies *
Wound healing (acute and chronic)	Reduce inflammation, support keratinocyte/fibroblast migration, enhance angiogenesis, and improve collagen organization	Adipose and umbilical cord MSC exosomes; dermal fibroblast and keratinocyte EVs; occasional platelet-derived EVs	Commonly NTA, TEM, and Western blot; some aesthetic series report only total protein or manufacturer data	Topical or perilesional use at debridement or surgery, with weekly repeats in some chronic ulcers
Photoaging and rejuvenation	Promote dermal remodeling; upregulate collagen and elastin; reduce oxidative and inflammatory signaling; support barrier repair	Adipose and umbilical cord MSC exosomes; cosmetic exosome blends; some plant-derived vesicles	Research-grade work generally reports NTA, TEM, and tetraspanins; many commercial products provide limited or no EV marker data	Applied after microneedling or fractional laser; often 3–4 sessions every 4–6 weeks.
Scars and procedural recovery (atrophic, hypertrophic, keloid)	Modulate fibroblast proliferation and myofibroblast transition; rebalance collagen I/III; adjust TGF-β/SMAD pathways; temper local inflammation	Adipose and perinatal MSC exosomes dominate the scar literature, including keloid and postsurgical models, while dermal fibroblast EVs and plant-based vesicle products appear in smaller cosmetic series focused on texture and pliability	Small clinical series often report basic size and particle counts, with variable use of Western blot for EV markers; most keloid and hypertrophic scar work is preclinical with full EV panels	Perioperative injection around the wound bed or topical use after fractional resurfacing; usually given once per procedure session, with follow-up procedures every 4–8 weeks when indicated
Alopecia and hair biology	Support dermal papilla–follicular stem-cell crosstalk; activate Wnt/β-catenin and PI3K–Akt signaling; reduce perifollicular inflammation and miniaturization	Dermal papilla exosomes; adipose and umbilical cord MSC exosomes; PRP-derived EVs; exploratory plant vesicles	Better-described studies report NTA with size distribution, TEM, and surface markers; several office series rely on manufacturer characterization only	Scalp injections given every 1–3 months; topical exosomes with microneedling weekly or every few weeks in 8- to 12-week courses
Inflammatory dermatoses (atopic dermatitis, psoriasis, acne)	Rebalance Th1/Th2 and Th17/Treg responses; decrease IL-4, IL-13, IL-17, TNF; restore barrier proteins (filaggrin, claudins); modulate sebocyte lipogenesis and TLR2 signaling	MSC exosomes; keratinocyte and fibroblast EVs; microbiome-derived EVs mainly studied as disease drivers	Preclinical and early translational studies typically include NTA, TEM, and Western blot panels; very few human reports, and commercial preparations rarely describe full EV characterization	Human data are limited; small atopic dermatitis studies use topical or intradermal preparations once or twice weekly for several weeks; there is no consistent dosing pattern yet
Skin infections and fungal disease	Pathogen EVs carry enzymes, toxins, and allergens that enhance virulence; plant and milk vesicles explored as carriers for antifungals and barrier-supporting vehicles	Pathogen-derived EVs from *Candida* and *Malassezia* (as mediators of disease); plant-derived vesicles (e.g., cucumber); bovine milk EVs	Pathogen EVs are defined mainly by proteomic and lipidomic profiles, whereas plant and milk vesicle carriers typically report NTA size, TEM morphology, and sparse marker data	Scheduling is undefined in humans; available preclinical studies use once-daily or intermittent topical plant or milk vesicles in skin models.

* The schedules are approximate, based on early clinical reports. No standardized maintenance regimen has been established.

## Data Availability

No new data were created or analyzed in this study. Data sharing is not applicable to this article.

## Abbreviations

The following abbreviations are used in this manuscript:

EV	Extracellular vesicle
DNA	Deoxyribonucleic acid
RNA	Ribonucleic acid
mRNA	Messenger RNA
miRNA	MicroRNA
NF-κB	Nuclear factor kappa B
STAT	Signal transducer and activator of transcription
TGF-β	Transforming growth factor beta
SMAD	Suppressor of mothers against decapentaplegic homolog
TIMP	Tissue inhibitor of metalloproteinases
MITF	Microphthalmia-associated transcription factor
PD-L1	Programmed death-ligand 1
PRP	Platelet-rich plasma
PRF	Platelet-rich fibrin
BMAC	Bone marrow aspirate concentrate
HA	Hyaluronic acid
MSC	Mesenchymal stromal cell (or mesenchymal stem cell)
MISEV	Minimal information for studies of extracellular vesicles
PBM	Photobiomodulation
LLLT	Low-level light therapy
OA	Osteoarthritis
AGA	Androgenetic alopecia
GMP	Good manufacturing practice
FDA	United States Food and Drug Administration
POSAS	Patient and Observer Scar Assessment Scale
CO_2_	Carbon dioxide (for fractional CO_2_ laser)
